# Polish Women Have Moderate Knowledge of Gestational Diabetes Mellitus and Breastfeeding Benefits

**DOI:** 10.3390/ijerph181910409

**Published:** 2021-10-03

**Authors:** Jolanta Lis-Kuberka, Magdalena Orczyk-Pawiłowicz

**Affiliations:** Department of Chemistry and Immunochemistry, Wroclaw Medical University, M. Skłodowskiej-Curie 48/50, 50-369 Wrocław, Poland; magdalena.orczyk-pawilowicz@umed.wroc.pl

**Keywords:** gestational diabetes mellitus, gestational programming, breastfeeding, child nutrition, human milk, public health, diabetes type 2, mother’s and newborn’s well-being

## Abstract

Gestational diabetes mellitus (GDM) is a multifaceted disease and is associated with complications for newborns and mothers. The aim of the study was to assess Polish women’s knowledge concerning GDM and their attitude to breastfeeding. As a research tool, an anonymous online survey that included 33 questions, grouped into three main sections—sociodemographic and obstetric variables, risk factors for GDM and neonatal adverse outcomes, and knowledge about breastfeeding—was used and administered online. A total of 410 women aged from 18 to 45 participated in this study. Based on the survey, it was demonstrated that the women had moderate knowledge concerning the maternal risk factors and adverse neonatal outcomes associated with GDM and, additionally, the short- and long-term effects of breastfeeding. Significantly deeper knowledge about GDM, including breastfeeding by GDM mothers, was observed among hyperglycemic mothers in comparison to normoglycemic mothers. However, knowledge concerning the health benefits of breastfeeding was not related to the mothers’ glycemic status. In conclusion, educational programs must include pre-pregnancy education of women and place emphasis on explaining the mechanism of development of GDM and the transformation of GDM to type 2 diabetes. This is crucial for changing the public’s perception of GDM as a temporary, reversible clinical entity.

## 1. Introduction

Gestational diabetes mellitus (GDM) is characterized as a disorder of glucose metabolism with the onset, or first appearance, occurring during pregnancy [1]. Normal pregnancy is diabetogenic due to the increased production of insulin (hyperinsulinemia) and insulin resistance (IR), in order to ensure adequate nutrition for the developing fetus [1,2,3,4,5]. Moreover, the increased concentration of hormones, such as estrogen, progesterone, and cortisol, as pregnancy progresses decreases the phosphorylation of insulin receptor substrate-1 (IRS-1), and may cause insulin resistance [2]; this is compensated by a higher production of insulin by beta cells of the islets of Langerhans. However, the pancreas’ capacity to produce insulin is not infinite, and finally this state leads to a gradual reduction of beta cell functioning and a decreased insulin level and may contribute to a glucose–insulin imbalance [3,6,7,8]. Moreover, increased maternal adipose tissue deposition, lower physical activity, and, additionally, higher food intake facilitate the development of relative glucose intolerance [3].

The peak effect of the hormonal changes (cortisol and estrogen) in pregnant women is observed between the 26th and 33rd week of gestation and this has become the indicator to perform basic screening tests for GDM (recommended period: 24th–28th week of gestation) [1]. The main criteria for the recognition of gestational diabetes mellitus are an abnormal fasting blood glucose level and/or an abnormal oral glucose tolerance test (OGTT) [9,10,11,12,13]. Unfortunately, the glucose level imbalance during pregnancy usually does not manifest with pronounced symptoms, so a diagnosis of diabetes for pregnant women is typically unexpected and raises many concerns related to care for the proper well-being of mother and a developed fetus.

The prevalence of GDM in Europe is estimated at 3–8% [14,15]. However, according to study groups of the World Health Organization (WHO) and the International Association of Diabetes and Pregnancy Study Groups (IADPSG), in certain populations, hyperglycemic disorders might affect more than 20% of pregnancies [16,17]. The basic approach to obtaining and controlling proper blood sugar levels is to follow a balanced diet and, additionally, to perform moderate daily physical activity, i.e., long walks, swimming or special activities for pregnant women (unless there are contraindications), which significantly impact proper weight gain during pregnancy and control ketonuria. Moreover, gestational diabetes mellitus might affect women’s health-related life quality and may contribute to the occurrence of anxiety, as well as a sense of loss of control over health in chronic diseases [18,19,20,21], and after delivery might contribute to a higher rate of postpartum depression [22].

The well-known established risk factors of GDM are advanced age (>35 years) [23,24], being overweight or obese [23,25,26], hypertension [27,28,29,30], a family history of diabetes [29,31], polycystic ovary syndrome (PCOS) [29,32], urbanization, a lack of physical activity, and a stressful environment [33,34]. On the other hand, pregnancy complicated by GDM is associated with an increased risk of complications for both mothers and newborns [35,36,37]. Maternal adverse outcomes include an increased incidence of pre-eclampsia/eclampsia [37], obstetric intervention [38], and the development of type 2 diabetes later in life [39,40]. Neonatal adverse outcomes of GDM include preterm delivery [41], macrosomia [42], postpartum hypoglycemia in the newborn [43], congenital malformations [44,45,46,47], glucose intolerance, excess weight and obesity, and type 2 diabetes mellitus in childhood and/or adulthood [48,49,50,51,52].

Currently, the high global prevalence of diabetes is a major public health problem. Nearly half a billion people have diabetes worldwide; the percentage of the population affected is projected to reach 25% in 2030, and even 51% in 2045 [34]. In the coming years, a change of mindset concerning gestational diabetes mellitus will be crucial. GDM is perceived as a temporary, reversible clinical entity, but it should be considered as a trans-generational, inherited metabolic disease due to the high rates of post-pregnancy conversion of gestational diabetes to type 2 diabetes [53], ranging from 2.6 to 70% in periods of 6 weeks to 28 years after pregnancy [40,54].

In Poland, the total number of births in 2020 was approximately 360,000. Assuming, on the basis of data from previous years, that about 3.6% of pregnant women develop gestational diabetes mellitus, this means that last year almost 13,000 women will have suffered from glucose imbalance [55]. The epigenetic changes related to glycemic imbalance (gestational programming) affect the health of the offspring [33,56,57,58]. The modified environment in utero of obese and diabetic mothers has an impact on the programming of fetal metabolic functions [33,58] and, eventually, on the health of the next generation. A clear understanding of the impact of GDM and its short- and long-term consequences for mothers and children by the government and public institutions will significantly contribute to controlling and slowing the progression of the diabetes epidemic [33,58]. Furthermore, it is imperative to strengthen the national strategy to support and promote breastfeeding.

Exclusive breastfeeding for the first 6 months of an infant’s life, with continued breastfeeding up to 2 years of age or beyond, is the gold standard recommended by the WHO [59]. Breastfeeding not only provides nutritional benefits but also plays an important role in early bonding and the long-term mental health and resilience of children [60,61]. Moreover, this natural way of feeding offspring decreases the risk of occurrence of infections in infants during the first 12 months of life, as well as the development of obesity and type 1 diabetes mellitus and allergies in children in later life [60,62,63]. The maternal benefits include the impact on post-partum weight loss, lowering the risk of the development of metabolic syndrome and type 2 diabetes mellitus in later life, and a lower incidence of breast and ovarian cancers [64,65].

Over the world, only 38% of infants aged 0 to 6 months are exclusively breastfed [66,67]. In Poland, at the end of the twentieth century, a high rate of initial breastfeeding (92%) was recorded; however, only 68% of mothers from the analyzed cohort breastfed exclusively [68]. In 2014, the breastfeeding rate for 6-week-old infants was 46% and rapidly decreased in subsequent months, reaching 17% and 11.9% at the ninth and twelfth months, respectively [60,69,70]. Moreover, the trend observed in the last five years in Poland is less optimistic, dropping dramatically with each subsequent month of lactation, reaching 28.9% at the fourth month and 4% in the sixth month [71]. One of the main reasons for giving up breastfeeding reported by mothers is milk deficiency, which is strictly associated with premature expansion of the infant’s diet and the appealing marketing of artificial milk [60,71]. However, according to [71,72], the dramatic reduction of breastfeeding is a net result of the lack of professional support and the recommendations of some experts and advisory committees, which are not fully aligned with the WHO. On the other hand, giving up breastfeeding is costly for mothers, due to the costs of formulas and accessories necessary for artificial nutrition, and, moreover, for public health, since breastfeeding prevents child and maternal diseases [73,74,75].

Considering the above, the aim of this study was to assess Polish women’s knowledge concerning gestational diabetes mellitus and their attitude to breastfeeding. Currently, there is a lack of reliable studies characterizing women’s state of knowledge about the impact of GDM on pregnant women and their children. Moreover, information regarding the level of knowledge about breastfeeding in relation to glycemic status among Polish mothers is also lacking.

## 2. Materials and Methods

### 2.1. Study Design

**Figure 1:**
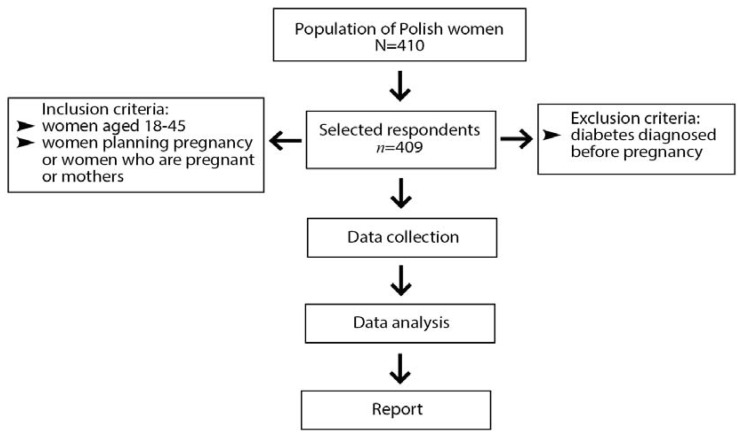
Scheme of women included in the study.

As a research instrument, a questionnaire (https://forms.gle/jrNG94p3dueMt8Bf9 (accessed on 10 Feburary 2021)) based on the research in the field was used. The survey was aimed at women aged 18–45 who were planning to become or who were pregnant, and women who were already mothers. Among the responders, one woman declared diabetes diagnosed before pregnancy and was excluded from the analysis (Figure 1).

### 2.2. Questionnaire Development

As a research tool, a 33-question anonymous, nationwide, and cross-sectional online survey assessing women’s knowledge of gestational diabetes mellitus and breastfeeding was used. The final questionnaire was divided into three main sections: (i) sociodemographic and obstetric variables, (ii) risk factors for gestational diabetes mellitus and neonatal adverse outcomes, women’s knowledge of gestational diabetes mellitus, and management of pregnancy, (iii) women’s knowledge of breastfeeding. Before starting the study the questionnaire was initially tested and the feedback was used to modify the final version. The questions were single- or multiple-choice, with a select few allowing for open-answer responses.

The reliability of the validated questionnaire was analyzed by determining Alpha Cronbach’s value, which ranged from 0.570 to 0.656. However, after removing the questions that were negatively correlated, the value of Alpha Cronbach reached a level ranging from 0.668 to 0.832. 

### 2.3. Data Source and Study Population

This research was conducted on the basis of a questionnaire approved by the Ethics Committee at Wrocław Medical University (No. KB-64/2021). The women were recruited from February 2021 to March 2021 using the survey, which was administered online using Google Forms, and potential participants gained access through local parenting groups that communicated via Facebook.

Based on the total number of births in 2020, and the prevalence of GDM in Poland, the estimated sample size was calculated using TIBCO STATISTICA ver. 13.3 (StatSoft, Inc., Tulsa, OK, USA) as 384 participants, with a level of confidence of 95% (alpha = 0.05) and a confidence interval of 5% (absolute ± %).

### 2.4. Variables

#### 2.4.1. Sociodemographic Variables

The independent sociodemographic variables (continuous variables) were age and pre-pregnancy body mass index (BMI). The ages of the respondents were reported by the women participating in the study were subsequently categorized as follows: 18–25, 26–30, 31–35, 36–40, and 41–45 years. The pre-pregnancy body mass index (BMI) was calculated after completing the survey, based on the body weight and height provided by respondents and classified according to the WHO guidelines [76]. The following categories were used: underweight (BMI below 18.5 kg/m^2^), normal weight (BMI ranging from 18.50 to 24.9 kg/m^2^), overweight (BMI ranging from 25.0 to 29.9 kg/m^2^), class- 1 obesity (BMI ranging from 30 to 34.9 kg/m^2^), class 2 obesity (BMI ranging from 35 to 39.9 kg/m^2^) and class 3 obesity (BMI above 40 kg/m^2^).

Moreover, the place of the respondents’ residence (>100,000 inhabitants, 10,000–100,000 inhabitants, <10,000 inhabitants and rural area), education level (primary, vocational, high school, and university), marital status (married, separated/divorced, cohabiting, single), monthly income per 1 person (<PLN 1500, PLN 1500–2999, PLN 3000–4500, and >PLN 4500), and the social status of the respondents were recorded. The social status, as independent data, was assigned according to the categories recommended by the Polish Economic Institute [77], namely class I—Higher grade professionals (doctors, professionals normally qualified with university degrees, teachers, directors and managers of companies), Class II—Lower grade professionals (medium-sized business owners, senior technicians, mid-level government administration, mid-level managerial staff), Class III—Routine non-manual employers and higher grade of administration and commerce, Class IV—business owners with employees, Class V—Business owners with employees, Class VI—Farm owners and self-employed in primary production, Class VII—Lower grade/level technicians (masters, foremen), Class VIII—Skilled workers, Class IX—Unskilled workers, and Class X—Agricultural workers.

#### 2.4.2. Obstetric Variables

The obstetric data included: current obstetric state/condition (pregnancy planning, first pregnancy, breastfeeding mother, and non-breastfeeding mother), parity, the gestational age of the last pregnancy (extremely preterm: 24–27 weeks, preterm 28–35 weeks, near term: 36–37 weeks, and term: 38–41 weeks), difficulty becoming pregnant (yes, no), and miscarriage in the past (yes, no).

#### 2.4.3. Women’s Knowledge concerning Gestational Diabetes Mellitus

The questions in this section of the survey included: the women’s glycemic status (normoglycemic or hyperglycemic), the presence of gestational diabetes mellitus (hyperglycemia compensated by diet (GDM G1), hyperglycemia compensated by diet and insulin treatment (GDM G2)), screening time for GDM (≤12 weeks of gestation, 13–23 weeks of gestation, recommended period (between 24 and 28 weeks of gestation), and after 28 week of gestation). Fasting glucose concentration (normal, abnormal) and glucose concentration (oral glucose tolerance test—OGTT) following the ingestion of 75 g of glucose after 1 h and 2 h (normal, abnormal) were determined according to declarations by the pregnant women during routine periodic visits, and the results were recorded in the patients’ pregnancy charts. Finally, the women were asked to assess their state of knowledge concerning gestational diabetes mellitus (using the scale: detailed, moderate, poor, and none) and sources of awareness of GDM (doctor, midwife, television/internet, books/parenting magazine, family members/friends, and education).

#### 2.4.4. Risk Factors for Gestational Diabetes Mellitus and Neonatal Adverse Outcomes

The data obtained from the survey in the area of maternal risk factors of GDM included: age above 35 years (yes, no), whether the mother was overweight or obese (yes, no), the presence of hypertension before pregnancy (yes, no), the occurrence of type 2 diabetes in a parent or sibling (yes, no), and polycystic ovary syndrome (yes, no).

The questions concerning the risk factors for newborns delivered by mothers with GDM included: higher risk of preterm delivery (yes, no), higher risk of fetal macrosomia (yes, no), higher risk of occurrence of postpartum hypoglycemia in the newborn (yes, no), higher risk of having a fetus or neonate affected by congenital anomalies (yes, no), higher risk of being overweight or obese in adulthood (yes, no), and higher risk of developing glucose intolerance and type 2 diabetes mellitus in adulthood (yes, no).

#### 2.4.5. Management of Pregnancy

The questions addressed to the respondents in the field of pregnancy management included: physical activity (yes, moderate, no), childbirth education (yes, no), consumption of supplements, folic acid, vitamin D and medication to treat allergies, upper respiratory tract infections, urinary and genital tract infections, thyroid disease, high blood pressure, and venous thromboembolism (yes, no) during pregnancy. The other pregnancy-related variables in the study were: consumption of alcohol (yes, occasionally, no), and smoking (yes, occasionally, no).

#### 2.4.6. Women’s Knowledge concerning Breastfeeding

The questions in this section of the survey included: declaration of breastfeeding by mothers (yes, no, I do not know), breastfeeding period (1 month, 3 months, 6 months, 7–12 months, >1 year, >2 years, not applicable). Moreover, the respondents were asked about the health benefits of breastfeeding for the baby and mother and the effects of early infant nutrition on growth and metabolism (metabolic programming).

#### 2.4.7. Assessment of Women’s Knowledge Level concerning GDM and Breastfeeding

The details concerning the assessment of knowledge levels were adapted from a study reported by Ramli et al. [78]. The following evaluation scale of grading, namely detailed (80–100%), moderate (60–79%), and poor (<60%) was used.

### 2.5. Statistical Analysis

The statistical analysis was performed with TIBCO STATISTICA ver. 13.3 (StatSoft, Inc., Tulsa, OK, USA). The manuscript contains a mixed data set (continuous and categorical variables). The continuous variables of the analyzed parameters (age, BMI) are given as the mean ± SD (standard deviation), whereas the categorical variables are presented as numbers and percentages (% (*n*/N)). The normality of the distribution in relation to the variables was checked with the Shapiro–Wilk test. Due to the abnormal distribution of the data, the chi-squared test was used to evaluate the differences between the groups in terms of categorical variables. Additionally, the Mann–Whitney U and Kruskal–Wallis tests were used for the continuous variables. A two-tailed *p*-value lower than 0.05 was regarded as significant. Univariate logistic regression was used to determine the predictors of the GDM knowledge levels, which showed *p* values lower than 0.05, using odd ratio (OR). The level of confidence was set at 95% with a probability level of *p* < 0.05 considered as statistically significant.

## 3. Results

### 3.1. Sociodemographic Variables

The mean age of the respondents was 30.8 ± 5.1 years and ranged from 18 to 45. More than half of the women (233/407; 57.2%) had a normal pre-pregnancy BMI and underweight, overweight and obese women constituted 6.4%, 22.4% and 14.0% of the total, respectively. The most numerous group consisted of mothers living in cities with a population of >100,000 (178/410; 43.4%), while 23.2%, 6.8% and 26.6% of the women stated that they lived in cities with a population 10,000–100,000, below 10,000, and in a rural area, respectively. Women who had graduated from university constituted the largest percentage of the study group (303/410; 73.9%). Most of the women were married (318/410; 77.6%), and single parenthood was reported by 2.9% of the respondents only (10/410) (Table 1).

In the study sample, more than a third of the women stated that they belonged to class III (152/400; 38.0%), the second most frequently mentioned social status was class VIII (79/400; 19.5%), and the third was class I (61/400; 15.3%). The most common monthly income per person reported by the women was 1500–2999 PLN (177/406; 43.6%).

The analysis of sociodemographic variables in relation to women’s knowledge level about GDM (detailed, moderate, poor, none) showed significant differences for: pre-pregnancy BMI (*p* = 0.02) (Table 2), place of residence (*p* = 0.03), marital status (*p* = 0.02), and monthly income per person (*p* = 0.02) (Table 2 and Table 3).

The detailed characteristics of the participants are shown in Table 1, Table 2 and Table 3.

### 3.2. Obstetric Variables

Among all the respondents who took part in the survey, the largest group (123/410; 30%) constituted women who were in their first pregnancy. This was followed by women who already had children but were not breastfeeding (104/410; 24.4%) or breastfeeding mothers (101/410; 24.6%). Women in a pregnancy that was not their first constituted 18.5% (76/410), while the respondents who were planning a pregnancy represented 1.5% (6/410) only.

Primiparous mothers accounted for nearly half of the study group (170/395; 43.0%) and the mean gestational age in the studied population was 39.2 ± 1.5 weeks, ranging from 35 to 42 weeks of gestation.

The majority of the respondents (300/407; 73.7%) reported having no difficulties in conceiving a child, although 18.1% (74/409) of the respondents had a miscarriage in the past (Supplementary Materials Table S1).

### 3.3. Management of Pregnancy

In the study sample, more than two-thirds of the women reported performing moderate physical activity during pregnancy (276/404; 68.3%), while no physical activity was reported by 10.9% (44/404). Moreover, 40.6% (165/400) of the respondents attended childbirth schools (Supplementary Materials Table S2).

The participants in the on-line survey stated that during pregnancy they had used supplements (365/396; 92.2%), including folic acid (366/385; 95.1%), and vitamin D (271/359; 75.5%). The women who suffered from allergies and upper respiratory tract infections during pregnancy were a definite minority: 4.8% (14/289) and 14.7% (44/299), respectively. Urinary and genital tract infections were reported by 33.2% (105/316) and 35.4% (111/314) of the pregnant women, while thyroid disease, high blood pressure, and venous thromboembolism were reported by 41.1% (136/331), 9.4% (28/299), and 17.3% (53/307) of the analyzed cohort of mothers, respectively. Alcohol consumption and smoking during pregnancy were reported by 3.2% and 6.9% of mothers, respectively (Supplementary Materials Table S2).

The analysis of the management of pregnancy in relation to maternal glycemic status showed significant differences for physical activity (*p* = 0.01) and the use of drugs to treat high blood pressure (*p* = 0.000008) and venous thromboembolism (*p* = 0.02) between the hyperglycemic and normoglycemic groups, but no differences for childbirth education, alcohol consumption and smoking were recorded (Table 4).

### 3.4. Women’s Knowledge concerning Gestational Diabetes Mellitus

In the analyzed cohort of mothers, the women with abnormal glycemic status during pregnancy constituted 35.7% (143/401) of the respondents and gestational diabetes mellitus compensated by diet was reported by 63.1% (89/141) of the women from the GDM group. The timing of the diagnosis of gestational diabetes, as reported by the women, was in the recommended period, i.e., between 24 and 28 weeks of gestation (53/140; 37.9%); however, 30.7% (43/140) of the respondents reported early GDM, which was diagnosed ≤12 weeks of gestation (Table 5).

The mean fasting glucose concentration in the GDM group was abnormal for 58.5% (64/109) of the pregnant women. On the other hand, 31% and 32% of the respondents demonstrated abnormal results on the oral glucose tolerance test (OGTT) following the ingestion of 75 g of glucose after 1 h and 2 h.

The overall level of knowledge concerning gestational diabetes mellitus was evaluated as moderate and poor for 47.5% and 35.0% of the respondents, respectively, and as the main source of knowledge about hyperglycemia during pregnancy, the women indicated: television/internet (216/398; 54.3%), doctors (205/398; 51.5%) and books/parenting magazines (108/398; 27.1%) (Table 5).

### 3.5. Risk Factors for Gestational Diabetes Mellitus and Adverse Neonatal Outcomes

The data obtained from the survey in the area of maternal risk factors for GDM showed that the respondents were familiar with the fact that the risk of the occurrence of GDM increased above 35 years old (210/396; 53.3%) as well as in the case of the mother being overweight or obese (319/402; 79.4%) (Supplementary Materials Table S3). Knowledge concerning the impact of the mother’s hypertension before pregnancy and type 2 diabetes in a parent or sibling on the occurrence of GDM was stated by 51.7% (202/391) and 66.0% (258/391) of respondents, respectively. The women who knew that polycystic ovary syndrome promotes the occurrence of GDM during pregnancy constituted only 31.3% (121/387) (Supplementary Materials Table S3).

The majority of the surveyed mothers were familiar with the neonatal adverse outcomes of gestational diabetes mellitus, such as preterm delivery (299/397; 75.3%), fetal macrosomia (324/403; 80.4%), congenital anomalies (225/388; 58.0%), postpartum hypoglycemia in the newborn (236/387; 61.0%), becoming overweight or obese in adulthood (242/390; 62.1%), and glucose intolerance and type 2 diabetes mellitus in adulthood (213/391; 54.5%) (Supplementary Materials Table S4).

**Figure 2:**
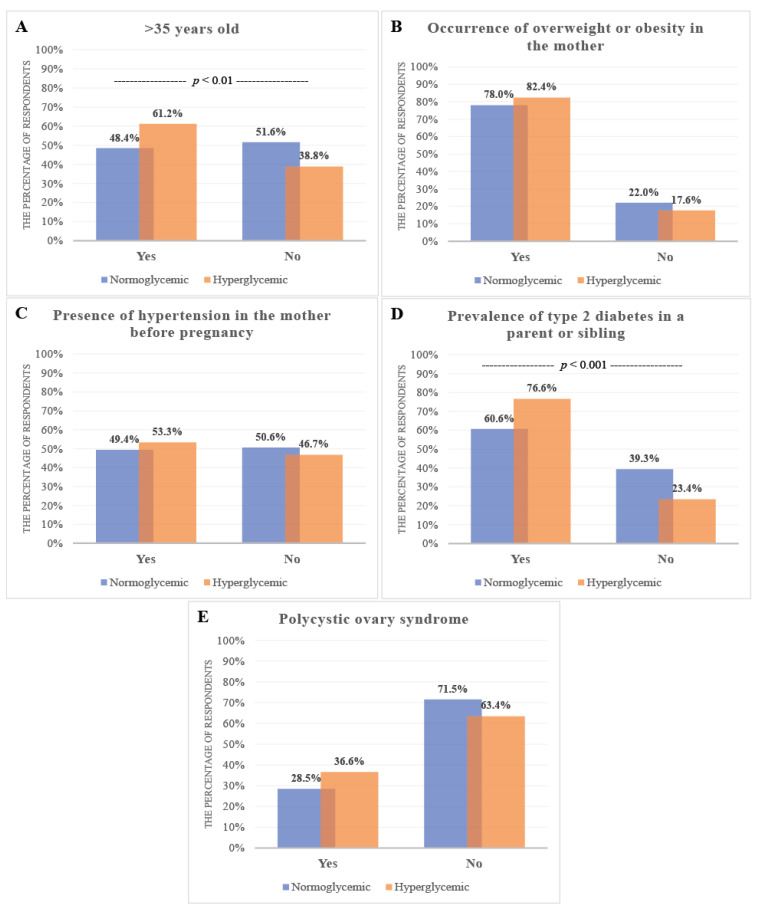
Women’s knowledge concerning the maternal risk factors for GDM in relation to maternal glycemic status. Knowledge levels among hyperglycemic and normoglycemic mothers. (**A**) Age of mother > 35 years old, (**B**) Excess weight or obesity in the mother, (**C**) Presence of hypertension in the mother before pregnancy, (**D**) Prevalence of type 2 diabetes in a parent or sibling, (**E**) Occurrence of polycystic ovary syndrome in the mother. The y axis expresses the percentage of women who said ‘yes’ or ‘no’.

**Figure 3:**
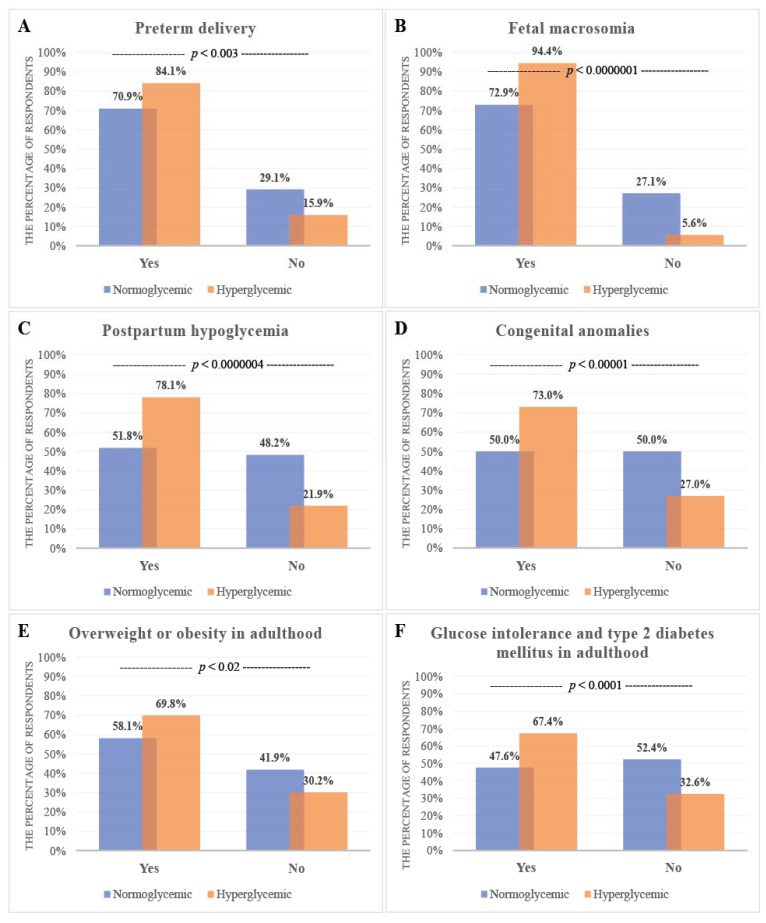
Women’s knowledge concerning adverse neonatal outcomes of GDM and in relation to maternal glycemic status. Knowledge levels among hyperglycemic and normoglycemic mothers. (**A**) Higher risk of preterm delivery, (**B**) Higher risk of fetal macrosomia, (**C**) Higher risk of postpartum hypoglycemia in the newborn, (**D**) Higher risk of having a fetus or neonate affected by congenital anomalies, (**E**) Higher risk of becoming overweight or obese in adulthood, (**F**) Higher risk of developing glucose intolerance and type 2 diabetes mellitus in adulthood. The y axis expresses the percentage of women who said ‘yes’ or ‘no’.

The analysis of women’s knowledge concerning maternal risk factors in relation to maternal glycemic status showed a significantly higher level in the hyperglycemic than the normoglycemic group concerning the risk associated with the mother’s age being above 35 years (*p* = 0.01) and the prevalence of type 2 diabetes in a parent or sibling (*p* = 0.001) (Supplementary Materials Table S5, Figure 2). The women’s knowledge concerning the adverse neonatal outcomes of GDM was significantly higher for the hyperglycemic group in comparison to the normoglycemic group with regards to the higher risk of occurrence of preterm delivery (*p* = 0.003), fetal macrosomia (*p* = 0.0000001), postpartum hypoglycemia (*p* = 0.0000004), congenital anomalies (*p* = 0.00001), becoming overweight or obese in adulthood (*p* = 0.02), and developing glucose intolerance and type 2 diabetes mellitus in adulthood (*p* = 0.0001) (Supplementary Materials Table S5, Figure 3).

### 3.6. Women’s Knowledge concerning Breastfeeding

In the present study, 92.4% (376/407) of the women declared their willingness to breastfeed. Among the women who had children, only 13.7% (55/402) and 15.7% (63/402) reported breastfeeding for over 1 and 2 years, respectively (Supplementary Materials Table S6).

The vast majority of (372/406; 91.6%) participants of the on-line study reported that they had been informed about the health benefits of breastfeeding for newborns/infants, i.e., strengthening emotional bonds (383/407; 94.1%), intellectual development (338/406; 83.3%); reduction of the risk of respiratory diseases (302/403; 74.9%), diabetes (243/403; 60.3%), and childhood obesity (264/401; 65.8%); and for the mother, i.e., reduction of the risk of breast (278/401; 69.3%) and ovarian (241/400; 60.3%) cancer (Supplementary Materials Table S6).

In total, 85% (347/408) of the respondents were familiar with metabolic programming in early life, and demonstrated a high level of knowledge concerning the possibility of breastfeeding by gestational diabetic mothers (351/407; 86.2%) (Supplementary Materials Table S6).

The analysis of breastfeeding knowledge levels in relation to maternal glycemic status showed a significantly higher level in the hyperglycemic group concerning the possibility of breastfeeding by a mother with diagnosed diabetes during pregnancy (*p* = 0.0001) (Table 6).

No statistically significant differences were found between breastfeeding knowledge levels in relation to age, BMI, education level, and monthly income per person of the women who participated in the study. However, the breastfeeding knowledge levels for specific types of information were related to the place of residence and the marital and social status of the respondents (Table 7). The place of residence was significant for only one out of the seven analyzed questions concerning breastfeeding (*p* = 0.02) (Have you been informed that breastfeeding reduces the risk of your baby developing diabetes?). By contrast, marital and social status were significant for four (*p* ranged from 0.0004 to 0.03) and two (*p* ranged from 0.001 to 0.01) out of seven analyzed questions, respectively.

### 3.7. Predictors of Good Knowledge of GDM

The univariate analysis identified that BMI (OR = 1.09; 95% CI = 1.05–1.14) significantly predicted a good level of GDM knowledge, while residing in an urban area with 10,000–100,000 residents (OR = 0.61; 95% CI = 0.35–1.07) and having a non-breastfeeding mother (OR = 0.59; 95% CI = 0.33–1.05) were significant predictors of a low level of GDM knowledge. By contrast, marital status and monthly income per person were not useful as predictors of the level of GDM knowledge (Table 8).

## 4. Discussion

According to scientific databases such as Web of Science, Scopus and PubMed, there are no reports characterizing in detail Polish women’s levels of knowledge concerning gestational diabetes mellitus and their attitudes towards breastfeeding. Moreover, these data are also not collected by the relevant government institutions dedicated to public health in the country. The present study attempted to deliver missing data essential for the promotion of and support for this important area of public health. The influence of socio-demographic data, obstetric variables, the management of pregnancy, the risk factors for gestational diabetes mellitus (including adverse maternal and neonatal outcomes), and women’s knowledge of gestational diabetes mellitus and breastfeeding were studied. Currently, the percentage of pregnancies affected by gestational diabetes mellitus is approximately 3–8% and the number of cases is systematically increasing [14,15]. The factors favoring the rapid development of diabetes include socio-cultural changes, aging societies, urbanization and modern lifestyles, i.e., the increased consumption of processed foods and carbohydrates, a significant reduction of the proportion of vegetables and fruit in the diet, and, finally, decreased physical activity [79,80,81,82,83,84].

To the best of our knowledge, the present survey-based study is the only such detailed analysis evaluating the level of knowledge concerning GDM among Polish women. However, Bień et al. [85] studied factors affecting the quality of life and acceptance of illness among pregnant women with diabetes, and Ługowska and Kolanowski [86] analyzed the nutritional behavior of pregnant women in Poland.

In the present study, a significant difference in the scores for knowledge about the adverse neonatal outcomes of GDM was found among participants with different glycemic statuses (Figure 3), with particular physical activity levels during pregnancy (*p* = 0.01), who used certain medications during pregnancy (*p* = 0.000008 for drugs to treat high blood pressure and *p* = 0.02 for drugs to treat venous thromboembolism), and with diabetes, who demonstrated particular levels of breastfeeding awareness (*p* = 0.0001). GDM knowledge levels (detailed, moderate, poor, none) were significantly different in relation to sociodemographic characteristics, such as pre-pregnancy BMI (*p* = 0.01), area of residence (*p* = 0.03), marital (*p* = 0.02), and economic status (*p* = 0.02), but not in relation to the age (*p* = 0.41), social status (*p* = 0.16), or educational level (*p* = 0.36) of respondents.

On the basis of the completed questionnaires, 47.5% of the participants declared that they had moderate and 10.5% detailed knowledge of the development of gestational diabetes. Among all the questions addressed to the respondents, the lowest knowledge level was observed for the maternal risk factors and adverse neonatal outcomes of GDM. In total, 51.6% of the normoglycemic participants and 38.8% (*p* = 0.01) of the hyperglycemic participants were not familiar with the fact that advanced maternal age is one of the risk factors of GDM. Additionally, more than 70% of the participants in the normoglycemic and more than 60% of the participants in the hyperglycemic group were unaware that polycystic ovary syndrome is also considered as a risk factor of GDM development (Figure 2).

Significantly lower knowledge levels about the adverse neonatal outcomes of GDM was found among normoglycemic mothers in comparison to hyperglycemic mothers, due to the greater education about diabetes among pregnant women with diagnosed GDM. The former were also much more aware that pregnancy affected by GDM leads to a higher risk of preterm delivery (84.1%), fetal macrosomia (94.4%), postpartum hypoglycemia in the newborn (78.1%), congenital anomalies (73.0%), overweight or obesity in adulthood (69.8%), and developing glucose intolerance and type 2 diabetes mellitus in adulthood (67.4%) (Supplementary Materials Table S5, Figure 3).

Based on the results collected from the section concerning women’s knowledge in the field of gestational diabetes mellitus, using the scale recommended by Ramli et al, [78], the overall data clearly indicate that Polish women have moderate knowledge levels (63.3%) concerning the risk factors for GDM (mean percentage of women in the normoglycemic (56.02%) and hyperglycemic (70.63%) group who answered ‘yes’ to the questions asked) (Supplementary Materials Table S5). In our opinion, the observed difference in knowledge levels concerning GDM between women with hyperglycemia and normoglycemia was related to the tendency among hyperglycemic women to search for additional information about disease, which allows them to understand and manage their condition. Our results are in line with the results presented by Park et al. The authors [87], who based their study on a questionnaire concerning knowledge and health beliefs about gestational diabetes and breastfeeding intention among Korean women with healthy pregnancies, concluded that education on breastfeeding for hyperglycemic women is extremely important and should focus on the benefits of breastfeeding and strengthening mothers self-efficacy.

Our findings indicate that more than 65% of our respondents performed moderate physical activity regardless of their glycemic status; however, a lack of physical activity during pregnancy was significantly more frequent among hyperglycemic Polish mothers (16.8%) in comparison to those who were normoglycemic (7.8%), which is in line with the latest research by Szatko et al. [88]. Pregnant women’s knowledge concerning physical activity seems satisfactory, but details concerning its health benefits, as well as the optimal frequency and duration, could be improved. It is interesting that almost one fifth of the study group of hyperglycemic women reported no physical activity during pregnancy, which suggests that the current status of the women (gestational diabetes) is not a sufficient incentive to take up physical activity.

In the survey, the respondents named television/internet (54.3%) as their main source of knowledge about gestational diabetes, as well as their obstetrician (51.5%), books/parenting magazines (27.1%), and family members/friends (24.9%). Unexpectedly, the midwife as a source of awareness of GDM was indicated by 14.3% of respondents only (Table 5). Midwives are responsible for preparing a mother for childbirth, and during visits they could make hyperglycemic mothers aware of the possible short- and long-term effects of gestational diabetes. Unfortunately, in many cases, gestational diabetes is considered a temporary disease, although research clearly shows that the conversion rate from gestational diabetes to diabetes is increasing [34,89]. Moreover, it should be noted that pregnant women with an impaired glucose level, which, according to the current criteria, is not considered as gestational diabetes, require dedicated glycemic monitoring as well as diabetic education.

The results presented in our study indicate that the encouragement of pregnant women to undertake dedicated physical activity by medical professionals is insufficient, despite the fact that it affects the well-being of the mother and the fetus. In light of the above, there is a need to reinforce the role of obstetricians and midwives as sources of reliable knowledge, which should be reflected in lowering the incidence of some complications during pregnancy. Additionally, as stated by Kwiendacz et al. [90], there is still a need to educate potential doctors in the field of gestational diabetes mellitus more thoroughly, since there are gaps in students’ knowledge related to diabetes. This indicates a need for persistent improvement in spreading diabetology knowledge during medical education in order to more efficiently combat the diabetes epidemic [90].

It should also be remembered that lowering a woman’s emotional anxiety may [91,92] result in better compliance and a better attitude towards changes of lifestyle, including diet and physical activity during and after completing pregnancy.

The reported frequency of maternal milk feeding in both the analyzed groups was very high, above 90%. However, the difficulties of the perinatal period and problems with stabilizing and sustaining lactation resulted in the abandonment of breastfeeding by 21.8% of normoglycemic mothers and 32.2% of hyperglycemic mothers within the first sixth months of lactation. Our results clearly show that there are still areas that could be improved relatively easily by the effectiveness of puerperium care as well as further prevention campaigns against the development of diabetes in the next generations.

In the analyzed cohort of Polish women, the breastfeeding knowledge level was related to some sociodemographic variables, namely marital and social status and place of residence. The identified differences are most likely related to limited breastfeeding education, especially in smaller localities (Supplementary Materials Figures S1 and S2). Unfortunately, access to qualified medical staff (e.g., lactation consultants) is limited and moreover often involves some additional costs that not all women can afford. The obtained data are in line with the results presented by Heck et al. [93], who demonstrated that socioeconomic status is a key predictor of a wide range of health behaviors and health outcomes among mothers. For this reason, the education of midwives and, potentially, neonatologists and pediatricians, who are all in direct contact with mothers, supporting and helping them through the difficult period of puerperium and the difficulties related to breastfeeding (e.g., mastitis, inflammatory breast diseases during lactation), play an extremely important role in this field.

The breastfeeding knowledge levels did not vary overall in relation to maternal glycemic status. According to the survey results, only one significant difference was found concerning breastfeeding by mothers with diagnosed gestational diabetes mellitus (*p* = 0.0001) (Table 6). In the group of normoglycemic mothers, almost 20% were not sure if mothers with diagnosed GDM could breastfeed their child, in contrast to less than 4% of hyperglycemic mothers. Based on the responses of the Polish women in both groups (normoglycemic and hyperglycemic), their breastfeeding knowledge levels according to the adopted knowledge scale were assessed as moderate independently of maternal glycemic status (69.51% and 79.14%, respectively). The results of our study clearly show that the hyperglycemic mothers were had more breastfeeding knowledge than normoglycemic respondents, and this might be explained by the fact that with the onset of gestational diabetes, they were inclined to seek information on the origins and management of this pathology.

Independently of their glycemic status, the mothers were familiar with nutritional programming. A child’s early-life metabolic programming can be considered a continuation of prenatal programming. Feeding mother’s milk as an exclusive form of nutrition up to 6 months is recommended for newborns by the WHO and is an important element of early-life metabolic programming, due to the associated long-term health benefits, such as protection against obesity and diabetes in later life [94,95]. In line with the above, breastfeeding is additionally important, because the occurrence of a glucose imbalance diagnosed for the first time during pregnancy (GDM) predisposes both the mother and the child to an increased risk of obesity and diabetes in later life. Early diagnosis of GDM, as well as efficient management, including correction by diet, physical activity, and dedicated education for diabetic mothers, may play an important role in decreasing the prevalence of diabetes and obesity. Hence, it has become a priority issue in public health [33].

In a global context, weak knowledge about the management of GDM was seen in Malaysian [96], Indian [97], Bangladeshi [98], and Nigerian [99] studies. These studies reported that most respondents were unfamiliar with the risk factors, screening time, and adverse maternal and neonatal outcomes of GDM. On the other hand, the findings of our study are in line with the results reported by Kaptein et al. [100], who demonstrated that healthcare providers should capitalize on hyperglycemic women’s motivation to make lifestyle changes during pregnancy to reduce their future risk of diabetes. Likewise, the meta-analyses published by Xu et al. [101] pointed out that among Chinese women, the benefits of GDM treatment/control are known, which reduces the possible consequences of GDM on the mother or the baby, while healthcare interventions aimed at GDM prevention are absent or extremely limited. A similar situation is observed in Poland.

In contrast to the data presented by Ogu et al. [99], our study revealed that place of residence and marital status did not predict levels of GDM knowledge. This was probably closely connected with the fact that, currently, the majority of women have access to information from different media. Among the analyzed variables, it was revealed that having a mother who did not breastfeed was a significant predictor of low levels of GDM knowledge. On the other hand, having been pregnant before or currently breastfeeding both related to the possibility of greater exposure to GDM information through attendance at antenatal and childbirth school, as well as to a greater likelihood of searching information about the wellbeing of mother and child in the perinatal and later periods. However, it should be pointed out that only conscious women or mothers are able to participate in some dedicated parental programs. Our findings showed that the provision of health education about GDM, especially for young women, even before the planned pregnancy, is extremally important. Our conclusions are in line with an observational study on a Finnish cohort by Laine et al. [102], who showed that information about the positive impact of breastfeeding, namely reducing the risk of the development of glucose intolerance and type 2 diabetes mellitus, on the later life of mothers and their children should be provided for young, poorly-educated, overweight women.

An unquestionable strength of the present study is that it included only women of a reproductive age, namely 18–45 years, which makes the studied cohort representative. Moreover, the data were derived from a national survey and obtained using a well-established, dedicated methodology, including a designed survey form and the calculation of a minimal sample size, all of which highlighted the problem of GDM in today’s society.

Some limitations should be considered when interpreting the findings of our study. Firstly, causal relationships between sociodemographic and obstetric variables, the management of pregnancy, and women’s knowledge concerning gestational diabetes mellitus and breastfeeding, could not be established due to the cross-sectional study design. Secondly, self-reported data such as the knowledge score concerning gestational diabetes mellitus and breastfeeding, were not validated using objective measures and may have been different between different participants’ responses. Additionally, the presented results did not take into account the emotional and psychological context of the women who participated in the study. Finally, it should also be noted that the use of an online survey may have an impact on the cohort analysis, due to variations in the cohort’s familiarity with the technology involved.

## 5. Conclusions

Our study revealed that the women whose responses we studied had a moderate level of knowledge about the maternal risk factors and adverse neonatal outcomes associated with GDM and, additionally, the short- and long-term effects of breastfeeding. Women’s understanding of abnormal glucose levels during and after pregnancy is extremely important as it allows the provision of modern diabetes therapy, which includes early prevention, identification and monitoring of risk factors. A conscious mother who understands her participation in the therapeutic process becomes an active partner in the control and supervision of GDM.

The results presented showed that the knowledge score for gestational diabetes mellitus and breastfeeding was moderate; thus, government agencies should increase efforts to educate society in the field of diabetes, childbirth education and the short- and long-term health benefits of breastfeeding, for both mothers and infants. The increasing prevalence of GDM prevalence and the high conversion rate from GDM to type 2 diabetes should prompt the government and public institutions to significantly contribute to controlling and slowing the progression of the diabetes epidemic, especially through early education.

The treatment of diabetes and its complications is expensive for both patients (due to the cost of anti-diabetic medication) and national economies (through the indirect costs incurred through loss of work, productivity or earnings) [81]. As reported by Wierzba et al. [103], in Poland spending on diabetes treatment is increasing steadily, and approximately 80% of diabetics in Poland state that they cannot afford optimal, modern diabetes therapy [104]. Therefore, the Polish diabetes policy should be specially aimed at a multidimensional approach and take into account a wide range of activities, including integrated programs at different levels, which is crucial for promoting healthy diets and daily physical activity.

## Figures and Tables

**Table 1:** Participants’ characteristics.

Sociodemographic Variable		*n*/N	%
Age (years)	18–25	56/409	13.7
	26–30	163/409	39.9
	31–35	113/409	27.6
	36–40	61/409	14.9
	41–45	16/409	3.9
Pre-pregnancy BMI, kg/m^2^	underweight (<18.5)	26/407	6.4
	normal weight (18.5–24.9)	233/407	57.2
	overweight (25–29.9)	91/407	22.4
	class 1 obesity (30–34.9)	41/407	10.1
	class 2 obesity (35–39.9)	12/407	2.9
	class 3 obesity (≥40)	4/407	1.0
Residence	urban, above 100,000 residents	178/410	43.4
	urban, 10,000–100,000 residents	95/410	23.2
	urban, <10,000 residents	28/410	6.8
	rural	109/410	26.6
Education	primary	1/410	0.2
	vocational	13/410	3.2
	high school	93/410	22.7
	university	303/410	73.9
Marital status	married	318/410	77.6
	divorced	5/410	1.2
	cohabiting	77/410	18.8
	single parent	10/410	2.4
Monthly income per person (PLN)	<1500	70/406	17.2
	1500–2999	177/406	43.6
	3000–4500	97/406	23.9
	>4500	62/406	15.3
Social status	Higher grade professionals	61/400	15.3
	Lower grade professionals	42/400	10.5
	Routine non-manual employers, higher grade of administration and commerce	152/400	38.0
	Small business owners with employees	11/400	2.8
	Small business owners without employees	18/400	4.5
	Farmers and small holders	8/400	2.0
	Lower grade technicians	9/400	2.3
	Skilled manual workers (outside agriculture)	78/400	19.5
	Unskilled workers (outside agriculture)	19/400	4.8
	Agricultural workers	2/400	0.5

The values shown in the table are given as the percentage of respondents in the given subgroup (*n*) in relation to all the respondents (N) for whom the specific information was available.

**Table 2:** GDM knowledge level in relation to age and BMI of respondents.

Sociodemographic Variable	Detailed N = 43 (% (*n*/N))	Moderate N = 194 (% (*n*/N))	Poor N = 143 (% (*n*/N))	None N = 28 (% (*n*/N))	*p*-Value
Age (years) (mean ± SD)	30.2 ± 4.2	31.2 ± 4.9	30.2 ± 5.4	31.6 ± 5.4	0.12
Pre-pregnancy BMI, kg/m^2^ (mean ± SD)	24.6 ± 5.2	24.9 ± 5.5	23.7 ± 4.2	21.7 ± 3.3	0.02

The values in the table are given as means ± SDs.

**Table 3:** GDM knowledge level in relation to sociodemographic characteristics.

Sociodemographic Variable		Detailed N = 43 (% (*n*/N))	Moderate N = 194 (% (*n*/N))	Poor N = 143 (% (*n*/N))	None N = 28 (% (*n*/N))	Chi-Square Test χ^2^	*p*-Value
Residence	urban, above 100,000 residents	49.0% (21/43)	47.4% (92/194)	39.2% (56/143)	25.0% (7/28)	18.06	0.03
	urban, 10,000–100,000 residents	14.0% (6/43)	18.6% (36/194)	28.7% (41/143)	42.9% (12/28)		
	urban, <10,000 residents	7.0% (3/43)	6.2% (12/194)	9.1% (13/143)	0.0% (0/28)		
	rural	30.2% (13/43)	27.8% (54/194)	23.1% (33/143)	32.1% (9/28)		
Education	primary	0.0% (0/43)	0.0% (0/194)	0.7% (1/143)	0.0% (0/28)	9.87	0.36
	vocational	0.0% (0/43)	2.1% (4/194)	4.9% (7/143)	7.1% (2/28)		
	high school	14.0% (6/43)	23.2% (45/194)	25.2% (36/143)	21.4% (6/28)		
	university	86.0% (37/43)	74.7% (145/194)	69.2% (99/143)	71.4% (20/28)		
Marital status	married	79.1% (34/43)	82.5% (160/194)	74.1% (106/143)	60.7% (17/28)	18.61	0.02
	divorced	0.0% (0/43)	0.5% (1/194)	2.1% (3/143)	3.6% (1/28)		
	cohabiting	20.9% (9/43)	16.5% (32/194)	18.2% (26/143)	32.1% (9/28)		
	single parent	0.0% (0/43)	0.5% (1/194)	5.6% (8/143)	3.6% (1/28)		
Monthly income per person (PLN)	<1500	4.7% (2/43)	14.6% (28/192)	23.4% (33/141)	25.0% (7/28	18.66	0.02
	1500–2999	55.8% (24/43)	44.3% (85/192)	41.8% (59/141)	25.0% (7/28		
	3000–4500	30.2% (13/43)	22.9% (44/192)	23.4% (33/141)	25.0% (7/28		
	>4500	9.3% (4/43)	18.2% (35/192)	11.3% (16/141)	25.0% (7/28		
Social status	Higher grade professionals	16.3% (7/43)	18.2% (35/192)	10.3% (14/136)	17.6% (5/28)	34.00	0.16
	Lower grade professionals	2.3% (1/43)	12.6% (24/190)	11.0% (15/136)	7.1% (2/28)		
	Routine non-manual employers, higher grade of administration and commerce	39.5% (17/43)	34.7% (66/190)	43.4% (59/136)	28.6% (8/28)		
	Small business owners with employees	4.7% (2/43)	1.6% (3/190)	2.9% (4/136)	7.1% (2/28)		
	Small business owners without employees	2.3% (1/43)	5.3% (10/190)	5.1% (7/136)	0.0% (0/28)		
	Farmers and small holders	0.0% (0/43)	3.2% (6/190)	1.5% (2/136)	0.0% (0/28)		
	Lower grade technicians	2.3% (1/43)	2.1% (4/190)	2.2% (3/136)	3.6% (1/28)		
	Skilled manual workers (outside agriculture)	30.2% (13/43)	18.4% (35/190)	16.2% (22/136)	25.0% (7/28)		
	Unskilled workers (outside agriculture)	0.0% (0/43)	3.2% (6/190)	7.4% (10/136)	7.1% (2/28)		
	Agricultural workers	0.0% (0/43)	0.5% (1/190)	0.0% (0/136)	3.6% (1/28)		

The values shown in the table are given as the percentage of respondents in the given subgroup (*n*) in relation to all the respondents (N) for whom the specific information was available.

**Table 4:** Women’s management of pregnancy in relation to maternal glycemic status.

Variable			Normoglycemic N = 258 (% (*n*/N))	Hyperglycemic N = 143 (% (*n*/N))	Chi-Square Test χ^2^	*p*-Value
Physical activity		Yes	22.5% (58/258)	17.5% (25/143)	8.17	0.01
		Moderate	69.8% (180/258)	65.7% (94/143)		
		No	7.8% (20/258)	16.8% (24/143)		
Childbirth education		Yes	40.7% (105/258)	41.3% (59/143)	0.01	0.91
		No	59.3% (153/258)	58.7% (84/143)		
Supplements and medications for pregnant women	Supplements	Yes	90.9% (231/254)	95.0% (133/140)	2.11	0.14
		No	9.1% (23/254)	5.0% (7/140)		
	Folic acid	Yes	95.2% (236/248)	95.6% (129/135)	0.03	0.86
		No	4.8% (12/248)	4.4% (6/135)		
	Vitamin D	Yes	77.5% (179/231)	72.2% (91/126)	1.23	0.27
		No	22.5% (52/231)	27.8% (35/126)		
	Drugs to treat allergy symptoms	Yes	5.7% (11/194)	2.2% (2/93)	1.80	0.18
		No	94.3% (183/194)	97.8% (91/93)		
	Drugs to treat upper respiratory tract infections	Yes	15.8% (32/203)	11.7% (11/94)	0.86	0.35
		No	84.2% (171/203)	88.3% (83/94)		
	Drugs to treat urinary tract infections	Yes	33.8% (70/207)	32.7% (35/107)	0.04	0.84
		No	66.2% (137/207)	67.3% (72/107)		
	Drugs to treat genital tract infections	Yes	34.1% (70/205)	37.4% (40/107)	0.32	0.57
		No	65.9% (135/205)	62.6% (67/107)		
	Drugs to treat thyroid disease	Yes	37.6% (80/213)	47.4% (55/116)	3.01	0.08
		No	62.4% (133/213)	52.6% (61/116)		
	Drugs to treat high blood pressure	Yes	4.1% (8/197)	20.0% (20/100)	19.74	0.00
		No	95.9% (189/197)	80.0% (80/100)		
	Drugs to treat venous thromboembolism	Yes	13.9% (28/202)	24.3% (25/103)	5.15	0.02
		No	86.1% (174/202)	75.7% (78/103)		
Alcohol consumption		Yes	0.8% (2/258)	0.7% (1/143)	1.11	0.58
		Occasionally	3.1% (8/258)	1.4% (2/143)		
		No	96.1% (248/258)	97.9% (140/143)		
Cigarettes/Smoking		Yes	3.9% (10/258)	1.4% (2/143)	2.57	0.27
		Occasionally	4.3% (11/258)	2.8% (4/143)		
		No	91.8% (237/258)	95.8% (137/143)		

The table shows the percentage of respondents in the given subgroup (*n*) in relation to all the respondents (N) for whom the specific information was available.

**Table 5:** Characterization of glycemic status of the analyzed cohort of mothers.

Variable		*n*/N	%
Glycemic status during pregnancy	Normoglycemic	258/401	64.3
	Hyperglycemic	143/401	35.7
Gestational diabetes treatment method	Hyperglycemia compensated by diet (GDM G1)	89/141	63.1
	Hyperglycemia compensated by diet and insulin treatment (GDM G2)	52/141	36.9
Timing of diagnosis of gestational diabetes mellitus	≤12 weeks of gestation	43/140	30.7
	13–23 weeks of gestation	33/140	23.6
	Recommended period, between 24 and 28 weeks of gestation	53/140	37.9
	After 28 week of gestation	11/140	7.9
Blood glucose concentration, tested while fasting	Normal: Abnormal:	45/109 64/109	41.3 58.7
Glucose tolerance test (OGTT)			
Glucose concentration after 1 h	Normal: Abnormal:	69/100 31/100	69.0 31.0
Glucose concentration after 2 h	Normal: Abnormal:	68/100 32/100	68.0 32.0
Women’s knowledge concerning gestational diabetes mellitus	Detailed	43/408	10.5
	Moderate	194/408	47.5
	Poor	143/408	35.0
	None	28/408	6.9
Sources of awareness of GDM	Doctor	205/398	51.5
	Midwife	57/398	14.3
	Television/internet	216/398	54.3
	Books/parental magazine	108/398	27.1
	Family members/friends	99/398	24.9
	Education	14/398	3.5
	Science publications	8/398	2.0

The data in the table show categorical values, which are given as the percentage of respondents in the given subgroup (*n*) in relation to all the respondents (N) for whom the specific information was available.

**Table 6:** Breastfeeding knowledge level in relation to maternal glycemic status.

Variable		Normoglycemic N = 258 (% (*n*/N))	Hyperglycemic N = 143 (% (*n*/N))	Chi-Square Test χ^2^	*p*-Value
Declaration of breastfeeding by mothers	Yes	91.9%5 (237/258)	93.7% (134/143)	0.60	0.74
	No	3.9% (10/258)	3.5% (5/143)		
	I do not know	4.3% (11/258)	2.8% (4/143)		
Breastfeeding period	1 month	5.4% (14/258)	11.7% (16/137)	10.79	0.09
	3 months	7.5% (20/258)	11.7% (16/137)		
	6 months	8.9% (23/258)	8.8% (12/137)		
	7–12 months	13.2% (34/258)	10.9% (15/137)		
	>1 year	16.3% (42/258)	9.5% (13/137)		
	>2 years	17.4% (45/258)	13.1% (18/137)		
	not applicable	3.1% (80/258)	34.3% (47/137)		
Have you been informed about the health benefits of breastfeeding for the baby and mother?	Yes	91.5% (236/258)	92.3% (131/142)	0.07	0.78
	No	8.5% (22/258)	7.7% (11/142)		
Do you think that a woman with diagnosed gestational diabetes can breastfeed her baby?	Yes	81.4% (210/258)	96.5% (137/142)	18.34	0.00
	No	1.9% (5/258)	0.0% (0/142)		
	I do not know	16.7% (43/258)	3.5% (5/142)		
Have you been informed that breastfeeding strengthens your emotional bonds?	Yes	93.4% (241/258)	95.8% (137/143)	0.97	0.32
	No	6.6% (17/258)	4.2% (6/143)		
Have you been informed that breastfeeding affects the intellectual development of the child?	Yes	81.4% (210/258)	86.6% (123/142)	1.79	0.18
	No	18.6% (48/258)	13.4% (19/142)		
Have you been informed that breastfeeding reduces the risk of respiratory diseases in a child?	Yes	75.2% (194/258)	75.0% (105/140)	0.00	0.97
	No	24.8% (64/258)	25.0% (35/140)		
Have you been informed that breastfeeding reduces the risk of your baby developing diabetes?	Yes	58.4% (150/257)	64.1% (91/142)	1.25	0.26
	No	41.6% (107/257)	35.9% (51/142)		
Have you been informed that breastfeeding reduces the risk of childhood obesity?	Yes	67.1% (171/255)	63.8% (90/141)	0.42	0.51
	No	32.9% (84/255)	36.2% (51/141)		
Have you been informed that breastfeeding reduces the risk of breast cancer in the mother?	Yes	67.2% (172/256)	73.6% (103/140)	1.74	0.18
	No	32.8% (84/256)	26.4% (37/140)		
Have you been informed that breastfeeding reduces the risk of maternal ovarian cancer?	Yes	59.8% (153/256)	61.9% (86/139)	0.17	0.68
	No	40.2% (103/256)	38.1% (53/139)		
Do you know that the nutrition of newborns and infants in the first 6 months of life has an impact on the child’s development in the later period (so-called metabolic programming)?	Yes	86.8% (224/258)	81.8% (117/143)	1.81	0.17
	No	13.2% (34/258)	18.2% (26/143)		

The values shown in the table are given as the percentage of respondents in the given subgroup (*n*) in relation to all the respondents (N) for whom the specific information was available.

**Table 7:** Breastfeeding knowledge levels in relation to sociodemographic variables.

	Sociodemographic Variable						
	Continuous Variables		Categorical Variables				
	Age	BMI	Residence	Education	Marital Status	Monthly Income Per Person	Social Status
Have you been informed that breastfeeding strengthens your emotional bonds?	*p* = 0.16	*p* = 0.66	χ^2^ 5.02 *p* = 0.17	χ^2^ 3.05 *p* = 0.38	χ^2^ 17.91 *p* = 0.00	χ^2^ 5.55 *p* = 0.13	χ^2^ 6.73 *p* = 0.66
Have you been informed that breastfeeding affects the intellectual development of the child?	*p* = 0.56	*p* = 0.62	χ^2^ 2.78 *p* = 0.42	χ^2^ 0.39 *p* = 0.94	χ^2^ 4.87 *p* = 0.18	χ^2^ 1.89 *p* = 0.59	χ^2^ 19.79 *p* = 0.01
Have you been informed that breastfeeding reduces the risk of respiratory diseases in a child?	*p* = 0.64	*p* = 0.81	χ^2^ 7.47 *p* = 0.06	χ^2^ 0.34 *p* = 0.95	χ^2^ 8.35 *p* = 0.03	χ^2^ 0.38 *p* = 0.94	χ^2^ 27.82 *p* = 0.00
Have you been informed that breastfeeding reduces the risk of your baby developing diabetes?	*p* = 0.46	*p* = 0.49	χ^2^ 9.44 *p* = 0.02	χ^2^ 1.94 *p* = 0.58	χ^2^ 4.27 *p* = 0.23	χ^2^ 1.06 *p* = 0.78	χ^2^ 8.59 *p* = 0.47
Have you been informed that breastfeeding reduces the risk of childhood obesity?	*p* = 0.43	*p* = 0.47	χ^2^ 4.90 *p* = 0.17	χ^2^ 0.66 *p* = 0.88	χ^2^ 8.54 *p* = 0.03	χ^2^ 2.16 *p* = 0.53	χ^2^ 15.40 *p* = 0.08
Have you been informed that breastfeeding reduces the risk of breast cancer in the mother?	*p* = 0.28	*p* = 0.66	χ^2^ 1.66 *p* = 0.64	χ^2^ 2.72 *p* = 0.43	χ^2^ 10.80 *p* = 0.01	χ^2^ 1.77 *p* = 0.62	χ^2^ 16.08 *p* = 0.06
Have you been informed that breastfeeding reduces the risk of maternal ovarian cancer?	*p* = 0.69	*p* = 0.61	χ^2^ 2.04 *p* = 0.56	χ^2^ 0.88 *p* = 0.83	χ^2^ 6.84 *p* = 0.07	χ^2^ 1.59 *p* = 0.66	χ^2^ 13.70 *p* = 0.13

**Table 8:** Predictors of good knowledge of GDM.

Variable	Odds Ratio (OR) (95% Lower—Upper Confidence Interval (CI))	*p*-Value
Pre-Pregnancy BMI	1.09 (1.05–1.14)	0.00
Residence		
Urban, above 100,000 residents (ref)		
Rural	1.33 (0.82–2.17)	0.09
Urban, <10,000 residents	1.07 (0.46–2.49)	0.73
Urban, 10,000–100,000 residents	0.61 (0.35–1.07)	0.03
Marital status		
Married (ref)		
Cohabiting	0.91 (0.54–1.54)	0.44
Single parent	0.51 (0.10–2.50)	0.68
Divorced	0.45 (0.05–4.05)	0.63
Obstetric condition		
A woman in her first pregnancy (ref)		
A woman who is planning to get pregnant	NA	-
Mother in subsequent pregnancy	1.38 (0.77–2.49)	0.12
Breastfeeding mother	1.32 (0.77–2.28)	0.14
Non-breastfeeding mother	0.59 (0.33–1.05)	0.00
Monthly income per person (PLN)		
1500–2999 (ref)		
<1500	1.18 (0.67–2.07)	0.12
3000–4500	0.77 (0.45–1.31)	0.60
>4500	0.58 (0.30–1.11)	0.10

NA—not analyzed, group size too small, ref—reference category.

## Data Availability

The data presented in this study are available on request from the corresponding author.

## Supplementary Materials

The following are available online at https://www.mdpi.com/article/10.3390/ijerph181910409/s1, Table S1. Obstetric variables, Table S2. Management of pregnancy, Table S3. Knowledge concerning maternal risk factors for gestational diabetes mellitus in analyzed cohort assessed based on questionnaire, Table S4. Knowledge concerning neonatal adverse outcomes of gestational diabetic mothers in analyzed cohort assessed based on questionnaire, Table S5. Women’s knowledge concerning maternal risk factors for GDM and neonatal adverse outcomes of GDM and in relation to maternal glycemic status, Table S6. Women’s attitude and knowledge concerning breastfeeding and short- and long-effects, Figure S1. Women’s knowledge concerning breastfeeding and in relation to sociodemographic variable: marital status (A-D), Figure S2. Women’s knowledge concerning breastfeeding and in relation to sociodemographic variables: place of residence (A) and social status (B and C).

## Abbreviations

BMI	body mass index
GDM	gestational diabetes mellitus
GDM G1	diet-controlled GDM
GDM G2	diet- and insulin-controlled GDM
IADPSG	International Association of Diabetes and Pregnancy Study Groups
OGTT	oral glucose tolerance test
PCOS	polycystic ovary syndrome
PGDM	pregestational diabetes mellitus
WHO	World Health Organization
