# Supplementary material for: Polish Women Have Moderate Knowledge of Gestational Diabetes Mellitus and Breastfeeding Benefits
Source: Int J Environ Res Public Health. 2021 Oct 3;18(19):10409. doi: 10.3390/ijerph181910409 (PMC8508017; doi:10.3390/ijerph181910409)
Supplement: Supplementary file 1 [file ijerph-18-10409-s001.zip › ijerph-1344017-supplementary.pdf]

International Journal of
Environmental Research and Public Health

MDPI

Supplementary Materials Table S1. Obstetric variables.

Variable		*n*/N	%
Current obstetric condition	A woman who is planning to get pregnant	6/410	1.5
	A woman in her first pregnancy	123/410	30.0
	Breastfeeding mother	101/410	24.6
	Non-breastfeeding mother	104/410	25.4
	Mother in subsequent pregnancy	76/410	18.5
Parity	0	123/395	31.1
	1	170/395	43.0
	2	76/395	19.2
	3	20/395	5.1
	≥4	6/395	1.5
Gestational age	A woman during her first pregnancy	46/123	37.4
	Breastfeeding mother	100/101	99.0
	Non-breastfeeding mother	103/104	99.0
	Mother in subsequent pregnancy	70/76	92.1
Did you have difficulties getting pregnant?	Yes	107/407	26.3
	No	300/407	73.7
Have you had a miscarriage in the past?	Yes	74/409	18.1
	No	335/409	81.9

The table shows the percentage of respondents in the given subgroup (*n*) in relation to all respondents (N) for whom the specific information was available.

Supplementary Materials Table S2. Management of pregnancy.

Variable			*n*/N	%
Physical activity		Yes	84/404	20.8
		Moderate	276/404	68.3
		No	44/404	10.9
Childbirth education		Yes	165/406	40.6
		No	241/406	59.4
Supplements and medications for pregnant women	Supplements	Yes	365/396	92.2
		No	31/396	7.8
	Folic acid	Yes	366/385	95.1
		No	19/385	4.9
	Vitamin D	Yes	271/359	75.5
		No	88/359	24.5
		Yes	14/289	4.8

	Drugs to treat allergy symptoms	No	275/289	95.2
	Drugs to treat upper respiratory tract infections	Yes	44/299	14.7
		No	255/299	85.3
	Drugs to treat urinary tract infections	Yes	105/316	33.2
		No	211/316	66.8
	Drugs to treat genital tract infections	Yes	111/314	35.4
		No	203/314	64.6
	Drugs to treat thyroid disease	Yes	136/331	41.1
		No	195/331	58.9
	Drugs to treat high blood pressure	Yes	28/299	9.4
		No	271/299	90.6
	Drugs to treat venous thromboembolism	Yes	53/307	17.3
		No	254/307	82.7
Alcohol consumption		Yes	3/405	0.7
		Occasionally	10/405	2.5
		No	392/405	96.8
Cigarettes/Smoking		Yes	13/404	3.2
		Occasionally	15/404	3.7
		No	376/404	93.1

The table shows the percentage of respondents in the given subgroup (n) in relation to all respondents (N) for whom the specific information was available.

Supplementary Materials Table S3. Knowledge concerning maternal risk factors for gestational diabetes mellitus in analyzed cohort assessed based on questionnaire.

**Variable**	**Yes** %, ***n*/N**	**No** %, ***n*/N**
Age > 35 years	53.3% (210/396)	47.0% (186/396)
Occurrence of overweight or obesity	79.4% (319/402)	20.6% (83/402)
Hypertension before pregnancy	51.7% (202/391)	48.3% (189/391)
Prevalence of type 2 diabetes in a parent or sibling	66.0% (258/391)	44.0% (133/391)
Polycystic ovary syndrome	31.3% (121/387)	68.7% (266/387)

The table shows the percentage of respondents in the given subgroup (*n*) in relation to all respondents (N) for whom the specific information was available.

Supplementary Materials Table S4. Knowledge concerning neonatal adverse outcomes of gestational diabetic mothers in analyzed cohort assessed based on questionnaire.

**Variable**	**Yes** %, *n*/N	**No** %, *n*/N
Higher risk of preterm delivery	75.3% 299/397	24.7% 98/397
Higher risk of fetal macrosomia	80.4% 324/403	19.6% 79/403
Higher risk of postpartum hypoglycemia in the newborn	61.0% 236/387	39.0% 151/387
Higher risk of having a fetus or neonate affected by congenital anomalies	58.0% 225/388	42.0% 163/388
Higher risk of overweight or obesity in adulthood	62.1% 242/390	37.9% 148/390
Higher risk of developing glucose intolerance and type 2 diabetes mellitus in adulthood	54.5% 213/391	45.5% 178/391

The table shows the percentage of respondents in the given subgroup (*n*) in relation to all respondents (N) for whom the specific information was available.

Supplementary Materials Table S5. Women's knowledge concerning maternal risk factors for GDM and neonatal adverse outcomes of GDM and in relation to maternal glycemic status.

**Variable**		**Normoglycemic** N = 258 (% (*n*/N))	**Hyperglycemic** N = 143 (% (*n*/N))	**Chi-square test** χ^2	***p*-Value**
Maternal risk factors for GDM					
> 35 years old	Yes	48.4% (122/252)	61.2% (85/139)	5.83	0.01
	No	51.6% (130/252)	38.8% (54/139)		
Occurrence of overweight or obesity in the mother	Yes	78.0% (199/255)	82.4% (117/142)	1.07	0.30
	No	22.0% (56/255)	17.6% (25/142)		
Presence of hypertension in the mother before pregnancy	Yes	49.4% (123/249)	53.3% (73/137)	0.53	0.46
	No	50.6% (126/249)	46.7% (64/137)		
Prevalence of type 2 diabetes in a parent or	Yes	60.6% (151/249)	76.6% (105/137)	10.13	0.00
	No	39.3% (98/249)	23.4% (32/137)		
Polycystic ovary syndrome	Yes	28.5% (71/249)	36.6% (49/134)	2.63	0.10
	No	71.5% (178/249)	63.4% (85/134)		
Neonatal adverse outcomes of GDM					
	**Yes**	70.9% (180/254)	84.1% (116/138)		

Higher risk of preterm	No	29.1% (74/254)	15.9% (22/138)	8.41	0.00
Higher risk of fetal macrosomia	Yes<br>No	72.9% (186/255)<br>27.1% (69/255)	94.4% (135/143)<br>5.6% (8/143)	27.05	0.00
Higher risk of postpartum hypoglycemia in the	Yes<br>No	51.8% (127/245)<br>48.2% (118/245)	78.1% (107/137)<br>21.9% (30/137)	25.54	0.00
Higher risk of having a fetus or neonate affected by congenital anomalies	Yes<br>No	50.0% (123/246)<br>50.0% (123/246)	73.0% (100/137)<br>27.0% (37/137)	19.13	0.00
Higher risk of overweight or obesity in adulthood	Yes<br>No	58.1% (143/246)<br>41.9% (103/246)	69.8% (97/139)<br>30.2% (42/139)	5.14	0.02
Higher risk of developing glucose intolerance and type 2 diabetes mellitus in	Yes<br>No	47.6% (118/248)<br>52.4% (130/248)	67.4% (93/138)<br>32.6% (45/138)	14.04	0.00

The table shows the percentage of respondents in the given subgroup (*n*) in relation to all respondents (N) for whom the specific information was available.

Supplementary Materials Table S6. Women's attitude and knowledge concerning breastfeeding and short- and long-effects.

Variable		*n*/N	%
Declaration of breastfeeding by mothers	Yes	376/407	92.4
	No	15/407	3.7
	I do not know	16/407	3.9
Breastfeeding period	1 months	32/402	8.0
	3 months	37/402	9.2
	6 months	35/402	8.7
	7-12 months	49/402	12.2
	>1 year	55/402	13.7
	>2 years	63/402	15.7
	not applicable	131/402	32.6
Have you been informed about the health benefits of breastfeeding for the baby and mother?	Yes	372/406	91.6
	No	34/406	8.4
Do you think that a woman with diagnosed gestational diabetes can breastfeed her baby?	Yes	351/407	86.2
	No	6/407	1.5
	I do not know	50/407	12.3
Have you been informed that breastfeeding strengthens your emotional bonds?	Yes	383/407	94.1
	No	24/407	5.9
Have you been informed that breastfeeding affects the intellectual development of the child?	Yes	338/406	83.3
	No	68/406	16.7
Have you been informed that breastfeeding reduces the risk of respiratory diseases in a child?	Yes	302/403	74.9
	No	101/403	25.1
Have you been informed that breastfeeding reduces the risk of your baby developing diabetes?	Yes	243/403	60.3
	No	160/403	39.7

Have you been informed that breastfeeding reduces the risk of childhood obesity?	Yes	264/401	65.8
	No	137/401	34.2
Have you been informed that breastfeeding reduces the risk of breast cancer in the mother?	Yes	278/401	69.3
	No	123/401	30.7
Have you been informed that breastfeeding reduces the risk of maternal ovarian cancer?	Yes	241/400	60.3
	No	159/400	39.7
Do you know that the nutrition of newborns and infants in the first 6 months of life has an impact on the child's development in the later period (so-called metabolic programming)?	Yes	347/408	85.0
	No	61/408	15.0

The table shows the percentage of respondents in the given subgroup (*n*) in relation to all respondents (N) for whom the specific information was available.

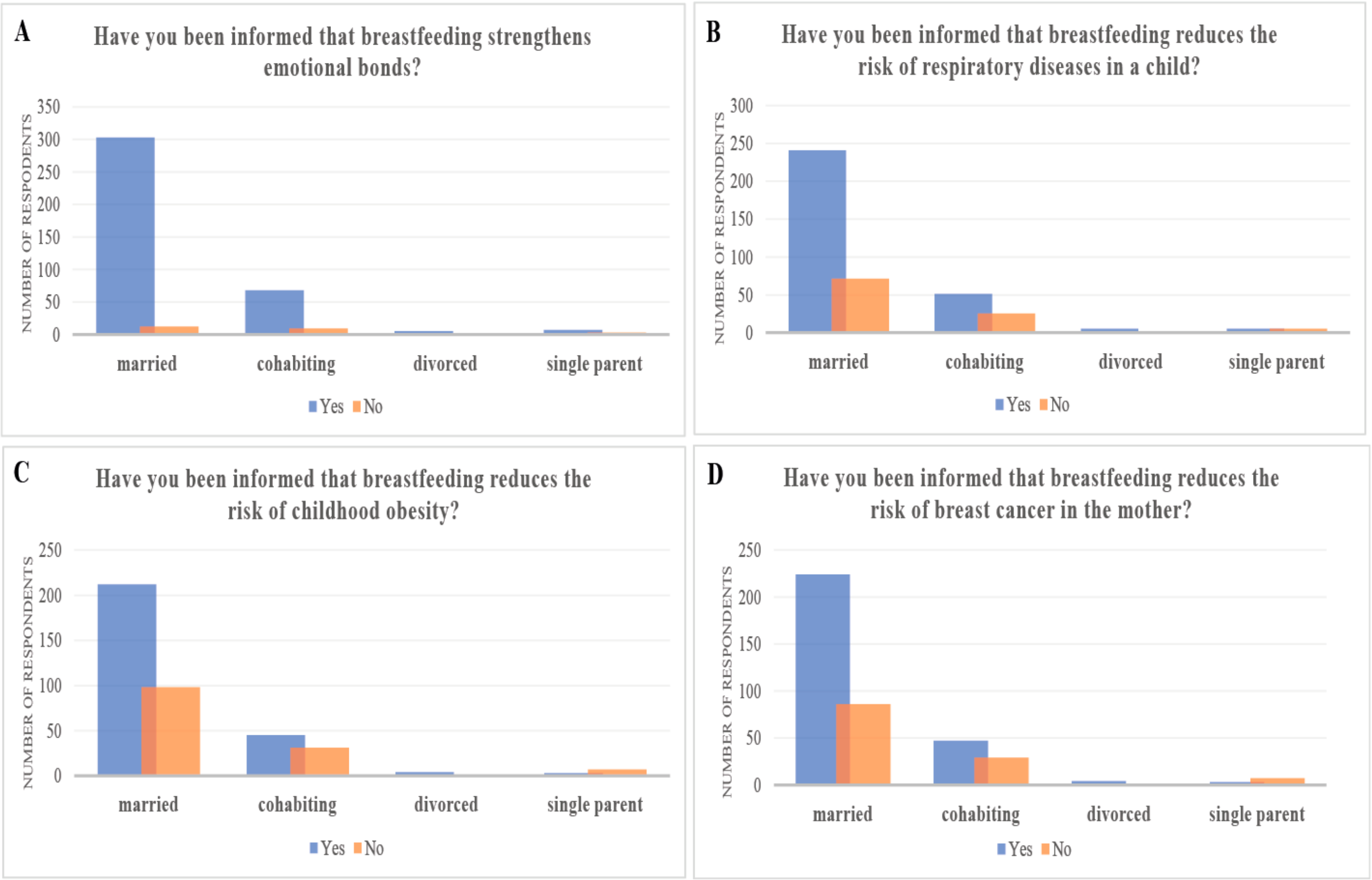

Supplementary Materials Figure S1. Women's knowledge concerning breastfeeding and in relation to sociodemographic variable: marital status (A-D).
The y axis expresses the percentage of women who said 'yes' or 'no'. The x axis expresses the sociodemographic variables.

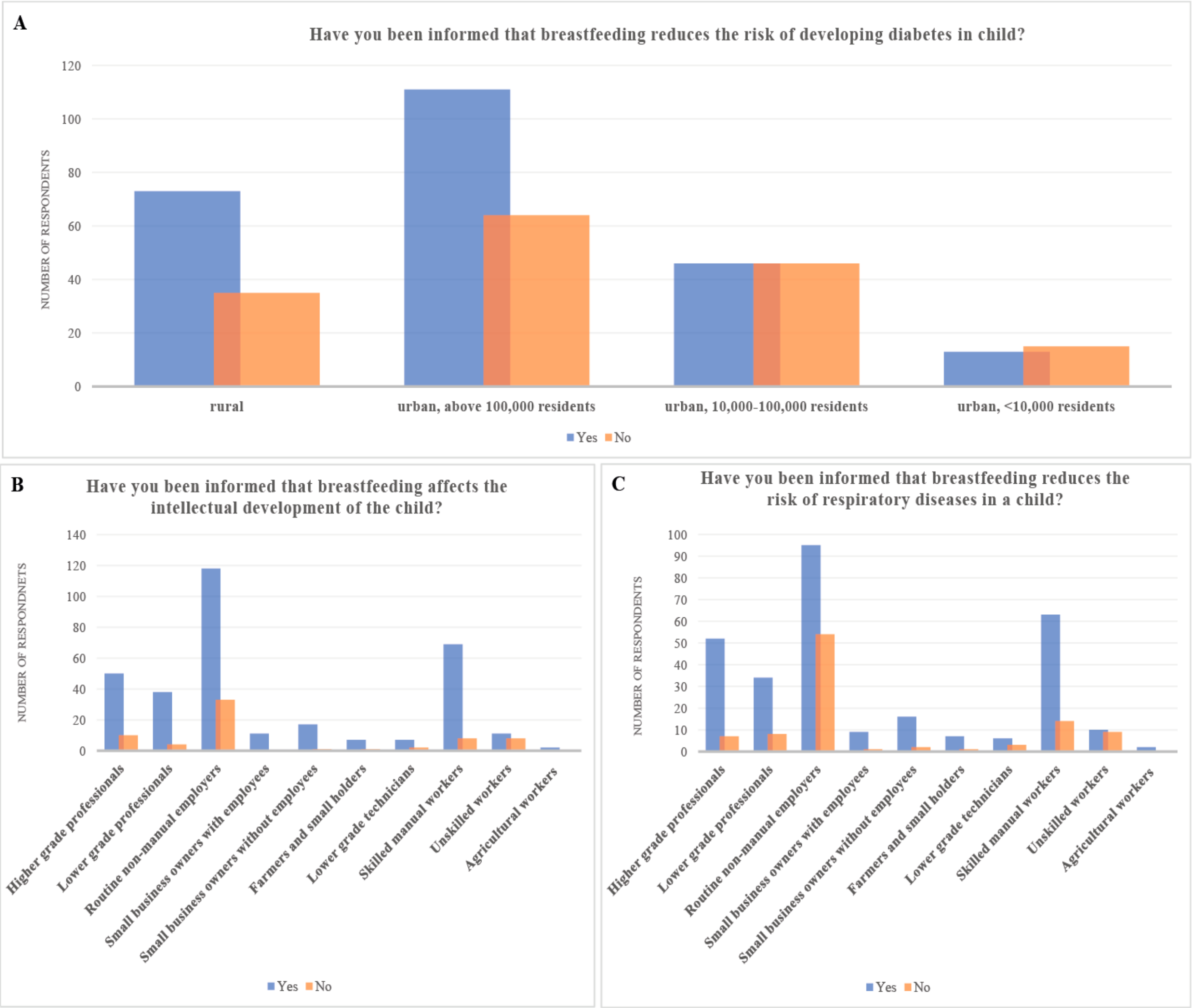

Supplementary Materials Figure S2. Women's knowledge concerning breastfeeding and in relation to sociodemographic variables: place of residence (A) and social status (B and C).
The y axis expresses the percentage of women who said ‘yes’ or ‘no’. The x axis expresses the sociodemographic variables.
